# Effects of rearing systems on the eggshell quality, bone parameters and expression of genes related to bone remodeling in aged laying hens

**DOI:** 10.3389/fphys.2022.962330

**Published:** 2022-08-31

**Authors:** Yu Fu, Jing Wang, Martine Schroyen, Gang Chen, Hai-jun Zhang, Shu-geng Wu, Bao-ming Li, Guang-hai Qi

**Affiliations:** ^1^ Key Laboratory of Feed Biotechnology, Ministry of Agriculture and Rural Affairs, Institute of Feed Research, Chinese Academy of Agricultural Sciences, Beijing, China; ^2^ Precision Livestock and Nutrition Laboratory, TERRA Teaching and Research Centre, Gembloux Agro-Bio Tech, University of Liège, Gembloux, Belgium; ^3^ Key Laboratory of Bio-environmental Engineering, Ministry of Agriculture and Rural Affairs, China Agricultural University, Beijing, China

**Keywords:** bone quality, eggshell quality, conventional caging system, laying hen, aviary system

## Abstract

Public concerns regarding animal welfare are changing the selection of rearing systems in laying hens. This study investigated the effects of rearing systems on eggshell quality, bone parameters and relative expression levels of genes related to bone remodeling in aged laying hens. A total of 2,952 55-day-old Jing Tint Six pullets were randomly assigned to place in the conventional caging system (CCS) or aviary system (AVS) and kept until 95 weeks of age. The AVS group delayed the decrease of eggshell quality and alleviated the symptoms of osteoporosis in the humerus rather than in the femur. Eggshell breaking strength, thickness, weight, weight ratio, stiffness and fracture toughness were decreased linearly with age (from 55 to 95 weeks of age, *p* < 0.05). The AVS group had higher eggshell breaking strength, stiffness and fracture toughness than the CCS group (*p* < 0.05). Higher total calcium and phosphorus per egg were presented in the AVS group at 95 weeks of age (*p* < 0.05). At 95 weeks of age, the AVS group had a humerus with higher weight, volume, length, midpoint perimeter, cortical index, fat-free dry weight, ash content, total calcium per bone, total phosphorus per bone, average bone mineral density, strength, stiffness and work to fracture compared to the CCS group (*p* < 0.05). Such differences did not appear in the femur. The relative expression levels of alkaline phosphatase (ALP) and osteocalcin (OCN) genes in the femur and hormone receptors (vitamin D receptor (VDR), estrogen receptor alpha (ERα) and fibroblast growth factor 23 (FGF23)) genes in the humerus were significantly upregulated (*p* < 0.05) in the AVS group. The level of tartrate-resistant acid phosphatase (TRAP) transcripts was also increased (*p* < 0.05) in the femur of the AVS group. Overall, compared with the CCS, the AVS alleviated the deterioration of eggshell and bone qualities of aged laying hens, which may be related to the changes in the expression of genes associated with bone remodeling.

## Introduction

Although the caging system has an edge in reducing the risk for feather-pecking, cannibalism and mortality (Fossum et al., 2009; Kjaer, 2009), the conventional caging system (CCS) limits the movement of hens and not welfare friendly to birds in aspects of the expression of natural behaviors (Bessei, 2019). In recent decades, the CCS has been prohibited in some regions (e.g. the European Union, Switzerland, Argentina, Australia, Brazil and New Zealand) through legislations and mandatory measures, but it still remains widespread in some countries such as China, Canada and the United States (Bessei, 2019). The transition from the CCS to the alternative or cage-free systems is an inevitable trend under multiple pressures from animal welfare and the international market scrutiny.

Aviary system (AVS), a cage-free system, is a promising alternation, which provides more activity space and enrichments (e.g., perches, terraces and platforms) to increase opportunities of locomotion, develop skills to navigate and reduce the risk of abnormal behavioral development (Hester, 2014; Janczak and Riber, 2015; Campbell et al., 2016). It not only improves animal welfare, but also has a positive impact on bone strengthening (Regmi et al., 2015). An early investigation in the causes of bone loss noted CCS had a higher incidence of osteoporosis (OP) (King, 1965) that is associated with fracture and premature mortality (Whitehead, 2004). Pullets raised in AVS has been evidenced to have bones with higher mineralization and strength (Regmi et al., 2015; Casey-Trott et al., 2017), and such benefits for bones could persist into the late period of laying (until the 14^th^ month of laying) (Leyendecker et al., 2005). Extended laying period (from 80 weeks of age to 100 weeks of age) is a commonly mentioned target in recent years (Pottgüter, 2016), which presents greater challenges for bone quality of laying hens, since the long-term mobilization of Ca related to eggshell formation may lead to the hen’s skeletal Ca to be absorbed into its uterus during eggshell formation, resulting in bone mineral loss and inducing OP (Wilson and Thorp, 1998; Cransberg et al., 2001). Additionally, the changes in bone quality in return could affect eggshell quality, as 20%–40% of the eggshell Ca content are derived from bone resorption (Comar and Driggers, 1949; Clunies et al., 1993). However, less attention has been given to the effects of rearing systems on eggshell quality. Our current study speculates that hens kept in AVS for a long time may alleviate the deterioration of bone and eggshell qualities compared to those kept in the CCS, which benefits the achievement of extended laying period. Thus, it is necessary to further compare the effects of CCS and AVS on eggshell and bone qualities of aged laying hens (over 80 weeks of age).

Given the complicated interactions between bone and eggshell, the mechanism of rearing systems on avian skeleton quality has been poorly understood. Two special types of skeletons remained during evolution in birds, one of which is a light but firm skeleton (without medullary bone) such as the humerus in charge of the ability to fly, and the other is a long bone containing medullary bone such as the femur (Dacke et al., 2015). The supply of Ca in eggshell formation depends on the rapid mobilization of medullary bone, while the humerus (mainly cortical bone) contributes only a little (Dacke et al., 1993; Dacke et al., 2015). Thus, the humerus and the femur may undergo bone remodeling in different manners. Their joint analysis may contribute to revealing the effects and molecular mechanisms of rearing systems on bone remodeling and eggshell formation.

This study tracked the eggshell quality of hens kept in the CCS and AVS at the late laying period, followed by analyzing their bone quality at the end of laying period. It then focused on the levels of bone remodeling-related transcripts and uterine Ca transport-related transcripts to explore the molecular mechanisms of rearing systems on bone remodeling and eggshell formation. This study may provide references for the selection of rearing systems in laying hens.

## Materials and methods

### Birds and experimental design

**FIGURE 1:**
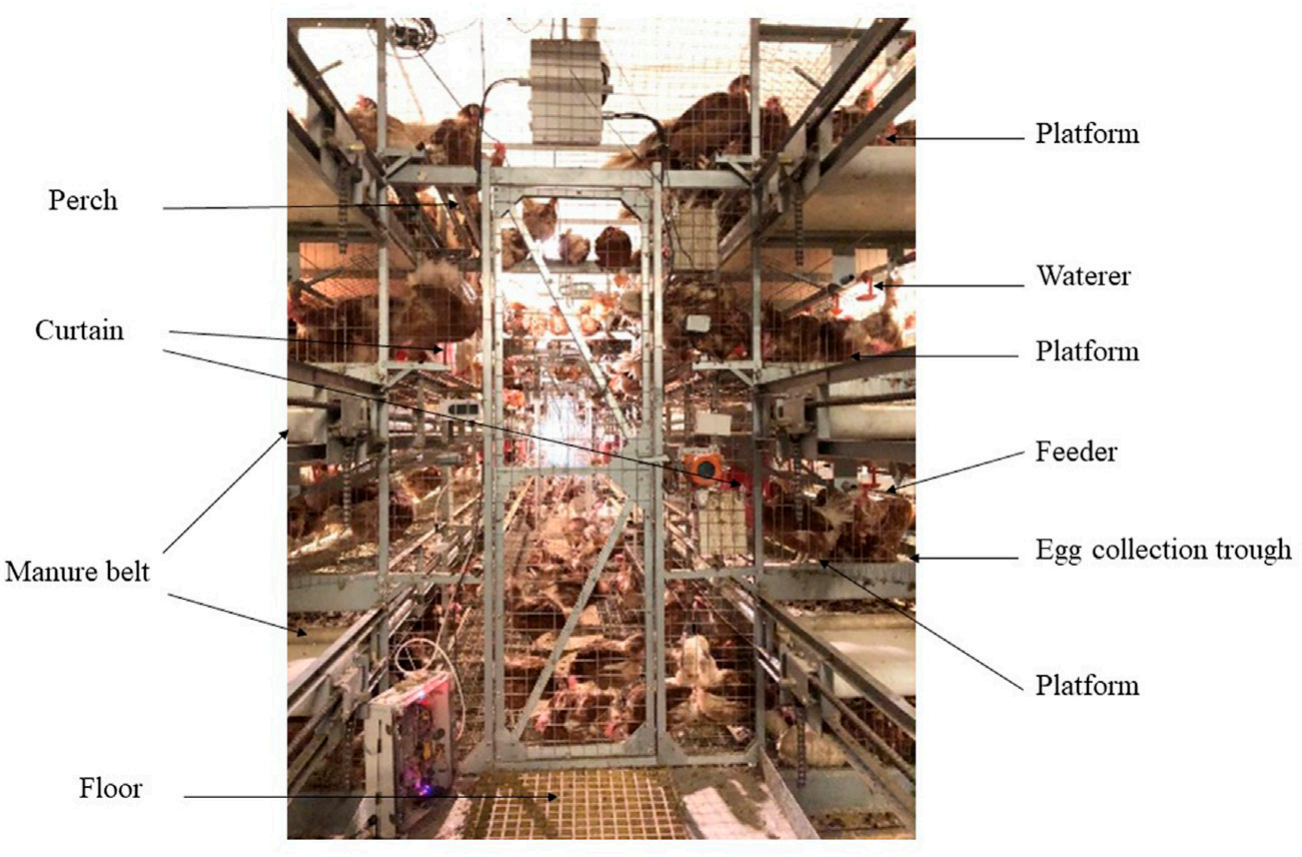
Image of aviary system employed in this study. The dimension (length × width × height) of the unit was 480 cm × 280 cm × 330 cm. Image depicts the main equipment inside the system (floor area 134,400 cm^2^, total system area 396,144 cm^2^), including feeders, waterers, perches, manure belts, litter area, nesting area and suspended platforms (261,744 cm^2^).

A total of 2,952 55-day-old Jing Tint Six pullets with similar body weight were purchased from Beijing Huadu Yukou Poultry Industry Co., Ltd. of China and randomly assigned to CCS and AVS. Jing Tint Six was based on Rhode Island Red and White Leghorn and featured with pink eggs. Breeder recommended, to 80 weeks, total egg number is around 380, the average egg weight is about 55.8 g, the egg production of the peak laying period is over 95%, the livability is more than 95%, and the average daily feed intake is around 106 g during laying period, and average body weight at 80 weeks is around 1800 g. The CCS was a four-tier battery system, whereas the AVS was a four-tier aviary system. Three hundred and sixty of hens were placed in 30 cages (12 hens per cage) of CCS with a space allowance of 450 cm^2^/hen; total cage area = 5,400 cm^2^. Another 2,592 hens were placed in 6 units of AVS with floor space allowance of 311 cm^2^/hen, and floor +3 layers suspended platforms space allowance of 917 cm^2^/hen (floor area 134,400 cm^2^, suspended platforms area 261,744 cm^2^, total system area 396,144 cm^2^). Each system was equally divided into six replicates, CCS had five cages per replicate and AVS had 1 unit per replicate. The dimension (length × width × height) of CCS cages and AVS units were 90 cm × 60 cm × 45 cm and 480 cm × 280 cm × 330 cm. As shown in Figure 1, the AVS had three levels of platforms above the floor, and each level contained two equally sized platforms on either side of the aviary unit interior. The perches of the same length as the platform were mounted on the upper platforms to increase opportunities for locomotion (Hester et al., 2014). Its nesting area was also installed in the platforms and separated by curtains. Each floor is equipped with the manure belt and egg collection trough. In the AVS, the floor and each level platform were equipped with feeders on both sides of the aviary unit, and waterlines with nipple waterers were suspended from the ceilings per level. The feeders and waterers of the CCS were fixed in the similar positions to the AVS.

The CCS and AVS were placed in one room of a research farm of China Agricultural University. This study was not terminated until the hens were 95 weeks of age. According to the nutrient requirements of the National Research Council (1994) and Chinese Feeding Standard of Chicken (Ministry of Agriculture of China, 2004), all pullets were fed a grower diet (CP = 15.5%, Ca = 0.8% and non-phytate *p* = 0.35%) until 18 weeks of age and changed to laying hen diets (19 weeks of age—the beginning of laying: CP = 17%, Ca = 2% and non-phytate *p* = 0.32%; the beginning of laying–55 weeks of age: CP:16.5%, Ca = 3.5% and non-phytate *p* = 0.32%; 55 weeks of age–95 weeks of age: CP = 15.5%, Ca = 3.8% and non-phytate *p* = 0.32%) later. All diets were purchased from a specialty feed mill. The detailed composition and nutrient levels of the diets were presented in the Supplementary Table S1. The analytical values of Ca and CP in diets from 55 to 95 weeks of age were 3.92% and 16.1%, respectively. All chickens were given feed and water *ad libitum* during the whole experiment. Two systems were given the same photoperiod (8–18 weeks of age: 9 h light/15 h dark, 15 lux; 18–30 weeks of age: increased stepwise to 16 h light/8 h dark, 30 lux; 30–95 weeks of age: 16 h light/8 h dark, 30 lux) and room temperature (15–23°C). The average daily egg production of the CCS and AVS for the whole laying period were 85.76% and 89.48%, respectively. And the average body weight at the end of trial was 1.894 kg in CCS and 1.752 kg in AVS.

### Sample collection

Each replicate (5 cages for CCS, 1 unit for AVS) was a sampling unit and an experimental unit. On the final day of 55, 65, 75, 85 and 95 weeks of age, all eggs from each replicate were collected individually, and 10 eggs of them were randomly selected to determine eggshell quality. During each time point, 60 eggs were examined for each system. At the end of the trial, two individuals were chosen randomly from each replicate to sacrifice. The mucosa samples of uterus and duodenum were immediately removed and snap-frozen in liquid nitrogen. Moreover, humeri and femurs on both lateral sides were dissected out and removed from meat and fat. The left bones were stored at −20°C for compositional, geometrical, morphological, or mechanical analyses. The right bones were truncated from their center. The proximal portion was immersed in formalin to fix. For the rest of the bone, a segment was cut in close proximity to the incision and snap-frozen in liquid nitrogen. Following collection, all samples frozen in liquid nitrogen were transferred to a freezer at −80°C until further analysis.

### Egg size and eggshell quality

Sixty eggs from each group were selected for testing at each time point (6 replicates with 10 eggs per replicate). Egg length and width were measured using caliper for calculation of shape index (egg shape index = egg length/egg width). The determination of eggshell quality referred to earlier described methods (Mabe et al., 2003; Fu et al., 2021a). Eggshell thickness was measured with Egg Shell Thickness Gauge (Ramat Hasharon, Israel Orka Food Technology Ltd., Ramat Hasharon, Israel). Eggshell breaking strength was defined as the minimum force required to fracture each egg at a longitudinal compression speed of 5 mm/min. It was measured with Egg Force Reader (Ramat Hasharon, Israel Orka Food Technology Ltd., Ramat Hasharon, Israel) at room temperature with the following parameters: the speed of the cross head, 5 mm/min; rated load capacity, 50 N; overload tolerance, 100 N; accuracy, 0.001 kgf. Eggshell weight was weighted as *w*_*1*_ after cleaning and drying in room temperature. The sum of dry weights of eggshells from each replicate was *W*_*1*_. The weight ratio of eggshell was calculated as a percentage score for the weight of each eggshell to the weight of each egg. For eggshell stiffness, a Texture Lab Pro (TMS-Pro, Food Technology Ltd., SV, Sterling, VA, United States) was used with the parameters as follow: the load of sensor, 25 N; the applied load, 10 N; the speed of the cross head, 1 mm/min. According to a previous report (Mabe et al., 2003), eggshell elastic modulus and fracture toughness were calculated as follow: the elastic modulus (N/mm^2^) = [(0.153 × I^3^)–(0.907 × I^2^) + (1.866 × I)–0.666]/0.444 × (0.408 + 3.026 × 2 × T/w) × (S_d_ × w/2/T^2^), the fracture toughness (N/mm^3/2^) = 0.777 × (2.388 + 2.9934 × 12/w) × F/T^3/2^, where I = egg shape index, T = eggshell thickness (mm), w = egg width (mm), Sd = eggshell stiffness (N/mm), F = breaking strength (N).

All eggshells from each replicate were mixed and crushed as one sample to analyze Ca and P contents according to the reported method (Fu et al., 2021b). Briefly, approximately 0.5 g of eggshell powder was taken and mixed with 3 mL nitric acid and 3 mL H_2_O_2_ in a burning cup and stood for 2 h. Then, the burning cup with eggshell was digested using a microwave digestion instrument (MDS-10, Shanghai Xinyi Instrument Technology Co., Ltd., Shanghai, China). After digesting, the solutions were transferred to 50 mL volumetric flasks and adjusted to 50 mL by rinsing 3 to 4 times deionized water. Flame atomic absorption spectrophotometry (Z2000, Hitachi Co., Ltd., Tokyo, Japan) was used to analyze the Ca content as *C*_*1*_. The content of P was measured as *C*_*2*_ with a spectrophotometer (UV-2700, Shimadzu, Japan). Total Ca and P per egg were calculated as follow: total Ca per egg = *W*_*1*_ *C_*1*_, total P per egg = *W*_*1*_ *C_*2*_.

### Bone geometric characteristics

Each group consisted of 6 replicates with 2 birds per replicate. The left bones were thawed at 4°C overnight before weighing. The volume was defined as the amount of water displaced when the bone was placed in a measuring cylinder with water. The bone density was the weight divided by the volume. A vernier caliper and a string were used to measure the length and the midpoint perimeter of the bone. The mean relative wall thickness, the cortical cross-sectional area and the mean cortical index were measured using the proximal portion of the right bone (fixed with formalin) and calculated according to previous methods (Brzoska et al., 2005; Cui et al., 2019). Briefly, the horizontal external and internal cortical bone diameters of the mid-diaphyseal cross section were measured as H and h using a digital caliper, and the vertical external and internal cortical bone diameter of that were measured as B and b. The mean relative wall thickness, the cortical cross-sectional area and the mean cortical index were calculated according to the following formula:

Mean relative wall thickness = [(B - b)/b + (H - h)/h]/2

Cortical cross–sectional area= π (H×B - h×b)/4

Mean cortical index = [(B - b)/B + (H - h)/H]/2

### Bone mineral measurements

Following the analyses of geometric characteristics, three regions of each left bone (6 replicates per group, each replicate based on 2 birds) were used for analyzing bone mineral density (BMD) and bone mineral content (BMC) using a dual energy X-ray absorptiometry system (DTX-200, Osteometer MediTech, Hawthorne, CA, United States). The bone was divided into three aliquots and marked with lead needles. The point located near from the body was considered as proximal, and the other was distal. Similarly, the midpoint of the bone was also marked with a lead needle. Bone segments 0.5 cm above and below the lead marker were used to measure BMD and BMC.

### Bone mechanical properties

After scanning, TMS-Pro was used to assess bone mechanical properties (strength, stiffness and work to fracture) using the three-point bending method reported by a previous study (Brzoska et al., 2005). Bone strength, stiffness and work to fracture were defined in the Table 1 according to the previous studies (Ferretti et al., 2001; Brzoska et al., 2005; Cui et al., 2019). Six replicates were used for each group and each replicate contains 2 birds. The span of two support bars was 4 cm. The load of sensor was 1,000 N. The bone was vertically loaded at a displacement rate of 2 mm/min until bone fracture.

**TABLE 1:** Definitions of the mechanical parameters.

Mechanical property	Definition
Bone strength	Minimum load to fracture the bone, descripting the resistance of the whole bone to fracture
Bone stiffness	The slope of the maximum elastic load-displacement curve, describing the resistance of the bone to deformation by a load
Work to fracture	The area under the load–displacement curve up to fracture, describing the amount of the total work done by imposed load to deform and fracture the bone or total energy absorbed by bone until fracture

### Bone components

Each group had 6 replicates and each replicate had 2 birds. The fracture bones were defatted completely and oven-dried at 65°C. The fat-free dry bone was weighed as *W*_*2*_ and then burned in a muffle furnace until they were fully burned to obtain bone ashes. The bone ash content was determined through calculating the percentage of the ash weight versus the fat-free dry weight. The Ca and P contents in ash were analyzed following the same method described above (Eggshell Quality). Total Ca and P in bones were calculated according to the following formulas: total Ca/P per bone = the Ca/P content in ash × (the ash content × *W*_*2*_).

### Bone histomorphometry

Histomorphological analysis was performed in a blinded manner. The method of Goldner’s Trichrome stain was adapted from a previous study (Farr et al., 2017). Femurs and humeri (6 replicates per group with 2 birds per replicate) were decalcified in EDTA until adequately softened. The softened bones were dehydrated, wax dipped, embedded and cut into 4-μm sections. The sections were deparaffinized and stained with Goldner staining solution suit (Servicebio technology Co. Ltd., Wuhan, China). Images were scanned with a panoramic slide scanner (3DHISTECH Ltd., Budapest, Hungary), and suitable areas of interest (AOI) in the proximal and middle parts of the bone were photographed with CaseViewer2.2 software (3DHISTECH Ltd., Budapest, Hungary). The AOI in proximal part of bone were defined as the entire epiphysis and metaphysis, and the AOI in the middle part of the bone was the mid-diaphyseal region (near the incision). Images of the epiphyseal region should contain the entire epiphyseal structure (including the outer cortical bone). Each image of metaphyseal and diaphyseal regions includes cortical bone on both sides. The regions were consistent across all bone samples. And, the same part was photographed at the same magnification.

The analysis of trabecular bone microarchitecture referred to a previous study (Lei et al., 2017) and was conducted with Image-Pro Plus 6.0 (Media Cybemetics, MD, United States) software. Tissue area (T.Ar), trabecular area (Tb.Ar) and trabecular perimeter (Tb.Pm) were measured. Trabecular number (Tb.N), trabecular thickness (Tb.Th), trabecular separation (Tb.Sp) and trabecular bone volume/tissue volume (BV/TV) were calculated using the following equations:

BV/TV = Tb.Ar / T.Ar × 100 (%)

Tb.Th = (2000 / 1.199) × (Tb.Ar / Tb.Pm) (μm)

Tb.N = (1.199 / 2) × (Tb.Pm / T.Ar) (n/mm)

Tb.Sp = (2000 / 1.19) × (T.Ar - Tb.Ar) / Tb.Pm (μm)

### Quantification of calcium metabolism-related mRNA

The tissue samples (including humerus, femur, uterus and duodenum; six replicates per group with 2 birds per replicate) were ground in liquid nitrogen first. EASYspin Plus Bone Tissue RNA Kit (Aidlab Biotechnologies Co. Ltd., Beijing, China) was used to extract total RNA in the humerus and femur. The total RNA in uterine and duodenal tissues were extracted using TRIzol reagent (Tiangen Biotech Co. Ltd., Beijing, China). The integrity, purity and concentration of RNA were determined by agarose gel electrophoresis and a NanoDrop 2000 spectrophotometer (Thermo Fisher Scientific, Waltham, United States). The cDNA synthesis was done on 1.5 μg total RNA with Easy Script First-Strand cDNA Synthesis SuperMix kit (TransGen Biotech Co. Ltd., Beijing, China). The resulting cDNA was used for subsequent quantification.

Quantitative real-time PCR (qPCR) was carried out by Light Cycler 480 system (Roche, Basel, Switzerland) with SuperReal PreMix kit (Tiangen Biotech Co. Ltd., Beijing, China). The results were processed using 2^−ΔΔCT^ method (Livak and Schmittgen, 2001). The sequences of primers were listed in Supplementary Table S2, the housekeeping gene used was avian β-actin.

### Statistical analysis

The replicate was the experimental unit. All analyses were conducted with SAS 9.4 (SAS Inc.). The homogeneity of variances and the normality of the data were tested first. The Proc GLM procedure was used to examine the effects of rearing system, age and the interaction between these two factors on the data of eggshell quality. The linear and quadratic orthogonal polynomial contrasts were further conducted for age response. Eggshell components, bone qualities and qPCR mean values of two rearing systems were compared using *t*-test procedure. Differences were considered statistically significant at *p* < 0.05.

## Results

### Egg size and eggshell quality

The results of egg size and eggshell quality are presented in Table 2. Egg length and egg shape index were linearly and quadratically increased with age (*p* < 0.05). The eggs in the AVS group had longer length and width compared to those in the CCS group (*p* < 0.05). Eggshell breaking strength, thickness, weight, weight ratio, stiffness and fracture toughness were decreased linearly with age (*p* < 0.05). Additionally, quadratic effects of age were detected on the egg weight, egg shape index, eggshell breaking strength, thickness, weight, weight ratio, stiffness and elastic modulus (*p* < 0.05). The AVS group had higher eggshell breaking strength, stiffness and fracture toughness than the CCS group (*p* < 0.05).

**TABLE 2:** Effect of rearing systems on eggshell quality of laying hens (55–95 weeks of age)^a^.

Item	Egg size			Eggshell quality						
	Length (mm)	Width (mm)	Shape index (mm/mm)	Breaking strength (N)	Thickness (*0.01 mm)	Weight (g)	Weight ratio (%)	Stiffness (N/mm)	Elastic modulus (N/mm^2^)	Fracture toughness (N/mm^3/2^)
System										
CCS	5.84^a^	4.30^a^	1.36	34.95^b^	43.42	5.60	9.16	66.74^b^	4,689.23	305.05^b^
AVS	5.79^b^	4.28^b^	1.35	36.57^a^	43.84	5.54	9.24	67.66^a^	4,643.77	316.65^a^
SEM	0.10	0.04	0.02	0.39	0.21	0.02	0.04	0.56	29.41	2.42
Age, wk										
55	5.69	4.29	1.33	37.57	44.30	5.62	9.49	72.83	4,898.44	317.54
65	5.75	4.28	1.34	38.61	45.28	5.67	9.47	70.98	4,566.53	317.52
75	5.87	4.29	1.37	35.20	43.64	5.54	9.07	65.67	4,552.50	306.25
85	5.85	4.28	1.37	35.23	43.09	5.53	9.10	64.10	4,571.99	313.60
95	5.91	4.31	1.37	32.17	41.82	5.50	8.86	62.42	4,743.05	299.33
SEM	0.10	0.04	0.02	0.39	0.21	0.02	0.04	0.56	29.41	2.42
*p*-value										
System	<0.001	0.028	0.424	0.002	0.145	0.150	0.166	0.029	0.337	0.011
Age										
Linear	<0.001	0.347	<0.001	<0.001	<0.001	0.007	<0.001	<0.001	0.144	0.017
Quadratic	<0.001	0.293	<0.001	<0.001	<0.001	0.027	<0.001	<0.001	<0.001	0.053
System × Age	0.785	0.319	0.476	0.477	0.270	0.871	0.430	0.796	0.089	0.385

a Data represent means with standard error mean. There were six replicates per age in each group. CCS, conventional caging system; AVS, aviary system.

^a,b^ Values within a column with no common superscripts differ significantly (*p* < 0.05).

**FIGURE 2:**
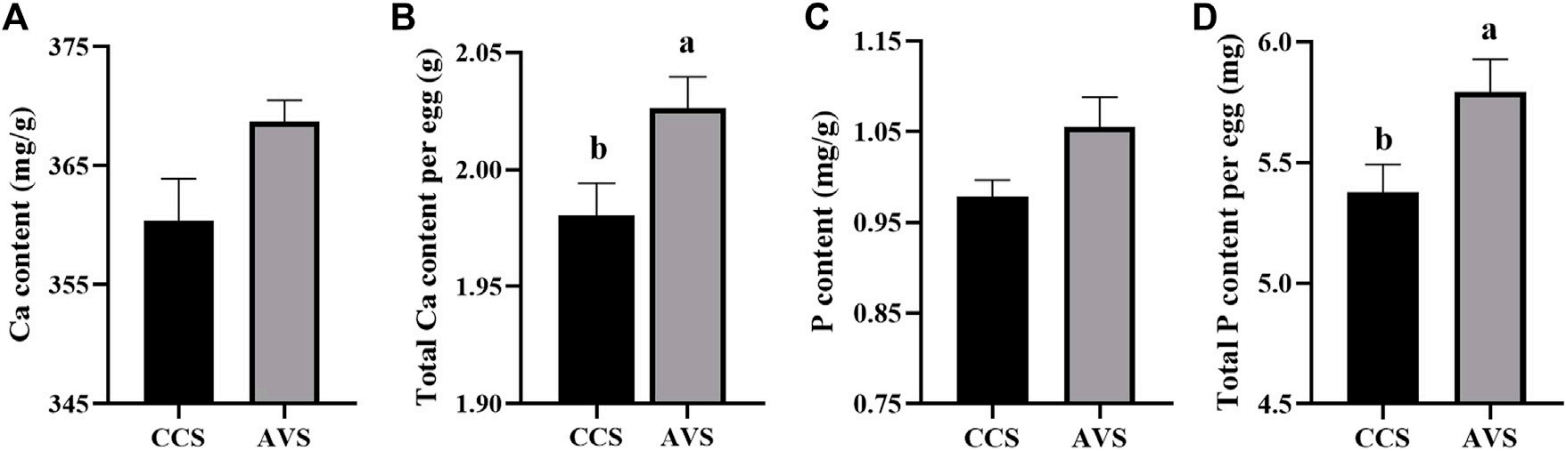
Effect of rearing systems on eggshell component of aged laying hens (95 weeks of age) (n = 6). **(A)** Ca content, **(B)** Total Ca per egg, **(C)** P content, **(D)** Total P per egg. CCS, conventional caging system; AVS, aviary system. a,b Bars with different letters differ significantly at *p* < 0.05.

In Figure 2, the results of eggshell component show that both total Ca and total P per egg in the AVS group were significantly higher than those in the CCS group (*p* < 0.05). In addition, eggshell Ca and P content were numerically higher in the AVS group compared to those in the CCS group, but the differences were not significant (Ca content, *p* = 0.071; P content, *p* = 0.063).

### Bone geometric characteristics, and components

**FIGURE 3:**
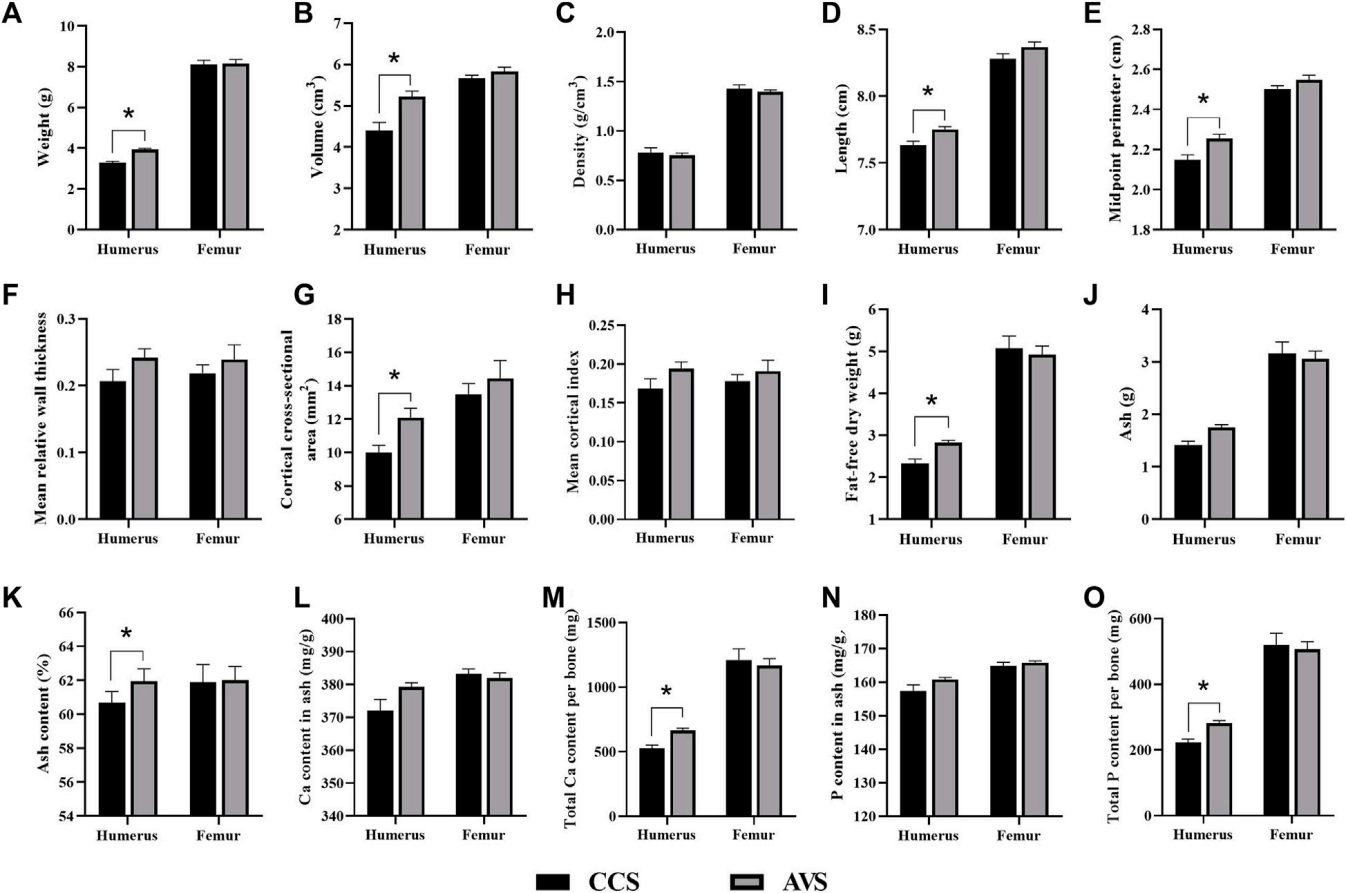
Effect of rearing systems on bone geometric characteristics **(A–H)** and component **(I-O)** in the aged laying hens (95 weeks of age) (n = 6). **(A)** Bone weight, **(B)** Volume, **(C)** Density, **(D)** Length, **(E)** Midpoint perimeter, **(F)** Mean relative wall thickness, **(G)** Cortical cross-sectional area, **(H)** Mean cortical index, **(I)** Fat-free dry weight, **(J)** Ash, **(K)** Ash content, **(L)** Ca content in ash, **(M)** Total Ca per bone, **(N)** P content in ash, **(O)** Total P per bone. CCS, conventional caging system; AVS, aviary system. Data represent means with standard error. *p < 0.05.

Figure 3 presents the differences in the geometric characteristics and components of the humerus and femur obtained from the AVS and CCS groups. Humeral weight, volume, length, midpoint perimeter and cortical index of the AVS group were obviously higher than those of the CCS group (*p* < 0.05). Compared to the CCS group, the AVS group had a humerus with higher fat-free dry weight, ash content, total Ca per bone and total P per bone (*p* < 0.05). There were no significant differences in the geometric characteristics and components of femur between the two groups (*p* > 0.05).

### Bone mineral measurements

Table 3 shows the comparative results of bone mineral measurements of laying hens. Compared to the CCS group, the AVS group had higher average BMD of the humerus (*p* < 0.05). The AVS group significantly increased distal BMD of the femur in comparison with the CCS group (*p* < 0.05).

**TABLE 3:** Effect of rearing systems on mineral measurements of bones in aged laying hens (95 weeks of age)^a^.

Items	CCS	AVS	*p-*value
Humerus			
Distal BMD (g/cm^2^)	2.77 ± 0.04	2.82 ± 0.04	0.344
Midshaft BMD (g/cm^2^)	2.81 ± 0.09	2.96 ± 0.04	0.141
Proximal BMD (g/cm^2^)	2.73 ± 0.03	2.80 ± 0.06	0.257
Average BMD (g/cm^2^)	2.77 ± 0.03^b^	2.86 ± 0.03^a^	0.042
Distal BMC (g)	2.50 ± 0.04	2.59 ± 0.06	0.268
Midshaft BMC (g)	1.62 ± 0.08	1.71 ± 0.04	0.296
Proximal BMC (g)	2.37 ± 0.03	2.49 ± 0.06	0.137
Average BMC (g)	2.16 ± 0.04	2.27 ± 0.05	0.134
Femur			
Distal BMD (g/cm^2^)	2.72 ± 0.03^b^	2.82 ± 0.02^a^	0.046
Midshaft BMD (g/cm^2^)	2.72 ± 0.09	2.87 ± 0.05	0.148
Proximal BMD (g/cm^2^)	2.77 ± 0.04	2.89 ± 0.07	0.154
Average BMD (g/cm^2^)	2.73 ± 0.04	2.84 ± 0.04	0.112
Distal BMC (g)	2.64 ± 0.04	2.71 ± 0.05	0.331
Midshaft BMC (g)	2.10 ± 0.06	2.23 ± 0.06	0.130
Proximal BMC (g)	2.62 ± 0.04	2.73 ± 0.08	0.238
Average BMC (g)	2.45 ± 0.04	2.56 ± 0.05	0.124

a Data represent means with standard error (n = 6). CCS, conventional caging system; AVS, aviary system; BMD, bone mineral density; BMC, bone mineral content.

^a,b^ Values within a row with no common superscripts mean significant difference (*p* < 0.05).

### Bone mechanical properties

As seen in the results summarized in Table 4, the strength, stiffness and work to fracture of the humerus in the AVS group were significantly higher than those in the CCS group (*p* < 0.05). However, these two groups did not differ significantly in femoral mechanical properties (*p* > 0.05).

**TABLE 4:** Effect of rearing systems on bone mechanical properties of aged laying hens[Table-fn Tfn3] (95 weeks of age)^a^.

Items	CCS	AVS	*p-*value
Humerus			
strength (N)	130.15 ± 6.09^b^	182.44 ± 9.50^a^	0.001
stiffness (N/mm)	67.67 ± 4.58^b^	92.01 ± 6.45^a^	0.012
work to fracture (mJ)	139.20 ± 11.47^b^	217.19 ± 14.88^a^	0.002
Femur			
strength (N)	270.43 ± 20.18	263.27 ± 16.06	0.787
stiffness (N/mm)	188.33 ± 23.99	152.59 ± 6.96	0.183
work to fracture (mJ)	241.41 ± 22.76	287.82 ± 25.15	0.201

a Data represent means with standard error (n = 6). CCS, conventional caging system; AVS, aviary system.

^a,b^Values within a row with no common superscripts mean significant difference (*p* < 0.05).

### Bone histomorphometry

**FIGURE 4:**
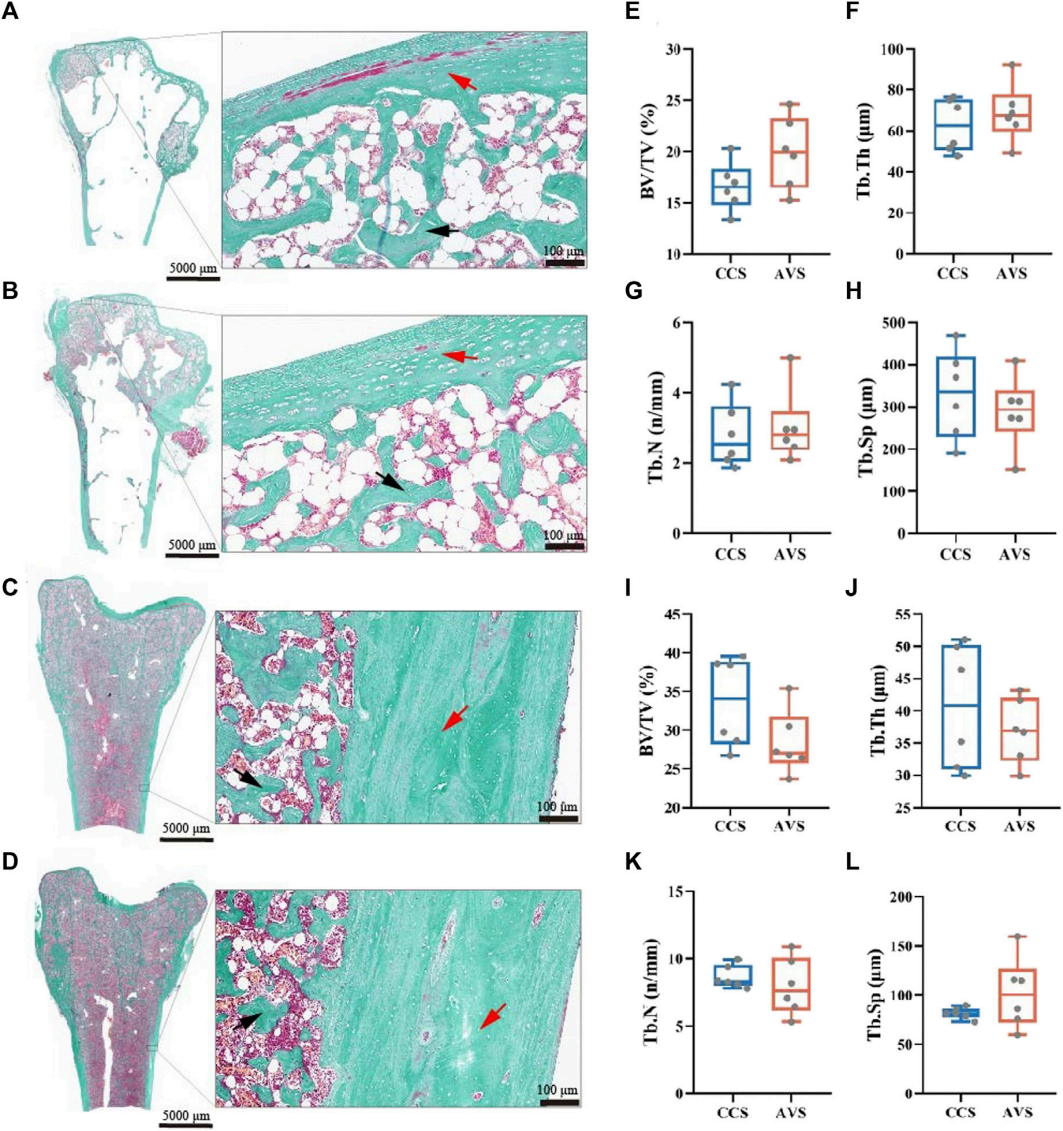
Effect of rearing systems on bone histomorphometry of aged laying hens (95 weeks of age) [humerus, **(A,B,E–H)**; femur, **(C,D,I-L)**]. Humeri and femurs were sectioned and stained with Goldner’s trichrome [**(A,C)**, the CCS group; **(B,D)**, the AVS group; red arrow, cortical bone; black arrow, trabecular bone]. **(E,I)** Trabecular bone volume/tissue volume (BV/TV), **(F,J)** Trabecular thickness (Tb.Th), **(G,K)** Trabecular number (Tb.N), **(H,L)** Trabecular separation (Tb.Sp). BV/TV, Tb.Th, Tb.N and Tb.Sp were counted under blinded analysis (n = 6). CCS, conventional caging system; AVS, aviary system.

The representative sections of the humeri and femurs stained with Goldner's Trichrome are shown in Figure 4 (A–D). The lateral margin of cortical bone was clear, while its medial edge lacked clear boundary with the adjacent trabecular bones. All humerus samples presented large cavities inside. In the humerus, most of trabecular bone and osteoid were distributed among the epiphysis, and a little in the metaphysis and diaphysis. The humerus of the AVS group had more trabecular bone and osteoid than that of the CCS group. The trabecular bone and osteoid of the femur were distributed throughout the bone cavity, accompanied by a few lacunae. Larger lacunae were observed in the femur of the AVS group compared to the CCS group. Figure 4 (E–L) shows the results of the blinded analysis of BV/TV, Tb.Th, Tb.N and Tb. Sp. There was a trend towards increased BV/TV (*p* = 0.085) in the humerus of the AVS group.

### Quantification of bone remodeling-related mRNA in bone

**FIGURE 5:**
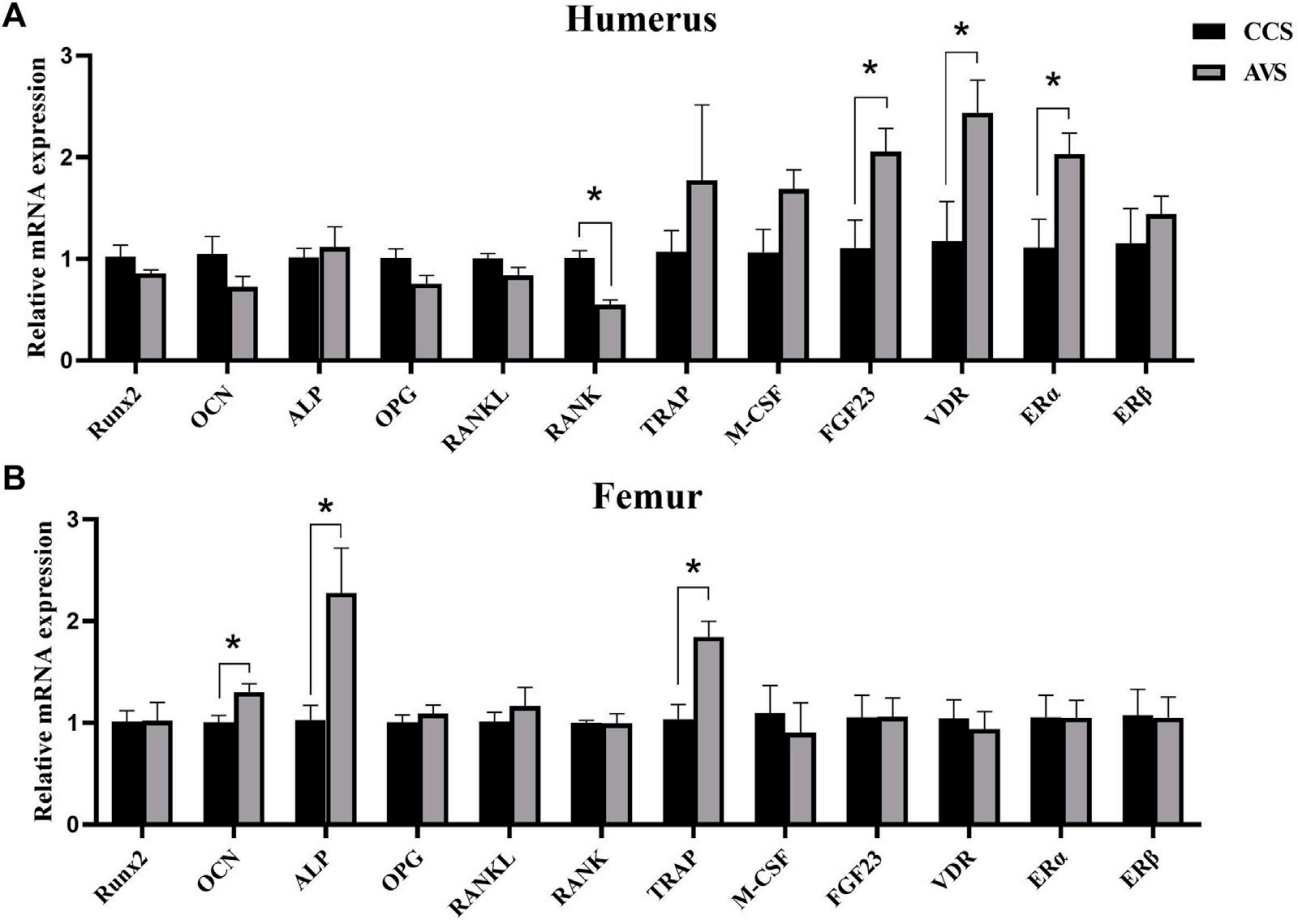
Effect of rearing systems on quantification of bone remodeling-related mRNA in bone of aged laying hens (95 weeks of age) (n = 6). **(A)** In the humerus, **(B)** in the femur. CCS, conventional caging system; AVS, aviary system. Runx2, runt-related transcription factor 2; OCN, osteocalcin; ALP, alkaline phosphatase; OPG, osteoprotegerin; RANKL, receptor activator of nuclear factor-kappa B ligand; RANK, receptor activator of nuclear factor-kappa B; TRAP, tartrate-resistant acid phosphatase; M-CSF, macrophage colony-stimulating factor; FGF23, fibroblast growth factor 23; VDR, vitamin D receptor; ERα, estrogen receptor alpha; ERβ, estrogen receptor beta. Data represent means with standard error. *p < 0.05.

The results of the expression levels of key genes involved in bone remodeling are shown in Figure 5. Compared to the CCS group, the AVS group significantly increased the relative mRNA expression levels of fibroblast growth factor 23 (FGF23), vitamin D receptor (VDR) and Estrogen receptor alpha (ERα) genes, but significantly decreased that of receptor activator of nuclear factor-kappa B (RANK) gene in humeri (*p* < 0.05). The relative mRNA expression levels of osteocalcin (OCN), alkaline phosphatase (ALP) and tartrate-resistant acid phosphatase (TRAP) genes in femurs were significantly upregulated in the AVS group compared to the CCS group (*p* < 0.05).

### Quantification of Ca transport-related mRNA in duodenum and uterus

**FIGURE 6:**
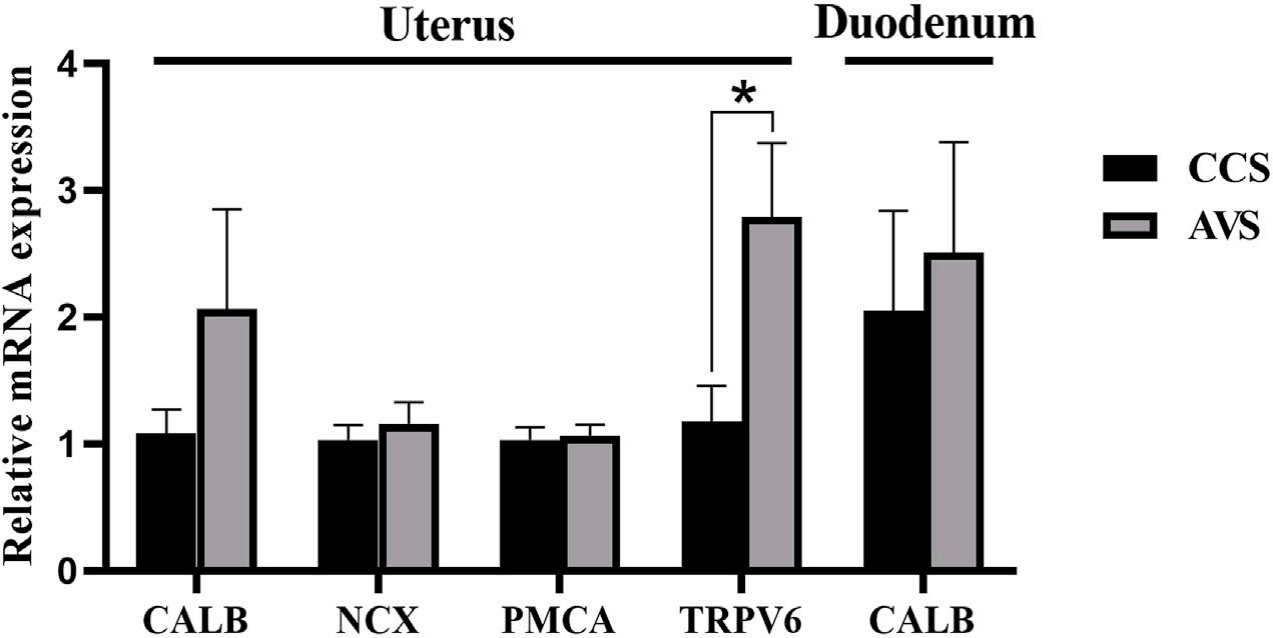
Effect of rearing systems on quantification of Ca transport-related mRNA in duodenum and uterus of aged laying hens (95 weeks of age) (n = 6). CCS, conventional caging system; AVS, aviary system. CALB, calbindin; NCX, Na^+^/Ca^2+^ exchange; PMCA, Ca^2+^ ATPase; TRPV6, transient receptor potential cation channel, subfamily V, member 6. Data represent means with standard error. *p < 0.05.

As seen in the results showed in Figure 6, no significant difference was observed in relative expression level of calbindin (CALB) in the mucosa of duodenum (*p* > 0.05). The AVS group significantly increased the transient receptor potential cation channel, subfamily V, member 6 (TRPV6) gene relative expression level in the mucosa of uterus compared to the CCS group (*p* < 0.05), while no significant effects were observed on the relative expression levels of CALB, Na^+^/Ca^2+^ exchange (NCX) and Ca^2+^ ATPase (PMCA) (*p* > 0.05).

## Discussion

Decreased eggshell quality in the late phase of laying poses a considerable threat to the economic benefit of poultry industry. Here, this study traced the eggshell quality of hens at the age of 55–95 weeks. In this stage, the egg size gradually increased and elongated as the age advanced, while eggshell breaking strength, thickness, weight, weight ratio, stiffness and fracture toughness exhibited a gradually decreasing trend, which well agreed with previous studies (Sirri et al., 2018; Gu et al., 2021). Notably, the AVS group seemed to delay these changes. Compared to CCS group, the AVS group had higher eggshell breaking strength, stiffness and fracture toughness. Previous report demonstrated that the AVS had eggshell with thicker thickness than CCS (Leyendecker et al., 2005; Ahammed et al., 2014). Thus, the hens that housed in AVS could produce high-quality eggshells. Calcium carbonate makes up about 96% of the eggshell and is a predominant contributor to its mechanical properties (Hincke et al., 2012). We found that total Ca and P per egg of the AVS group higher than those of the CCS group. This demonstrated that Ca involved in eggshell formation was more in AVS group, and the increase of Ca supply may be a reason of the enhancement in eggshell mechanical properties (Fu et al., 2021a).

During bone remodeling, more bone formation and less bone loss increased bone strength and reduced the incidence of bone fracture (Whitehead, 2004). The AVS group exhibited higher weight, volume, length, midpoint perimeter, cortical index, fat-free dry weight, ash content, total Ca per bone and total P per bone in the humerus, indicating the AVS group had benefits in bone development and remodeling compared to the CCS group. The ultrastructure and mineral measurements of bones are the primary means of diagnosing OP (Brandi, 2009; Rachner et al., 2011). The average BMD of humerus and the distal BMD of femur were significantly increased in the AVS group compared to the CCS group. Similarly, there was a trend (*p* = 0.085) towards increased BV/TV in the humerus. Although no objective diagnostic parameters of OP existed in laying hens, the humerus of the CCS group tilted towards OP symptoms (Brandi, 2009) compared to that of the AVS group. The most severe consequence of OP is fracture, which is the leading cause of osteoporosis-related mortality. A three-point bending test was performed in our current study to simulate the process by which a bone was damaged by force. The strength, stiffness and work to fracture of the humerus were lower in the CCS group, indicating the hens held in this group may be susceptible to fracture in routine activities or accidental injuries. The increase of mechanical properties in the AVS group may be attributed to the enhancements of bone ultrastructure and bone mineral measurements, since the increase of trabecular bone connectivity and mineral deposition offered higher bone fracture resistance (Chen et al., 2020). Previous studies confirmed that exercise played roles in enhancing bone mineral density and ultrastructure (Zymbal et al., 2019), and preventing OP in elder people (Feskanich et al., 2002). These benefits were also confirmed in pullets housed in AVS, which were equipped with perches and platforms to increase opportunities for locomotion (Regmi et al., 2015; Casey-Trott et al., 2017). Our study demonstrated that such effects would carry over to the end of laying phase, but only in the humerus and not in the femur, since similar differences were not found in the femur. Similar results were also observed in a previous study, wherein hens housed in the CCS and AVS had no significant differences on the ash and Young’s Modulus of the tibia (Regmi et al., 2016). The marrow cavity of the femur contains medullary bone, which is a source of Ca for eggshell formation (Nys and Le Roy, 2018). The AVS group with high-quality eggshells had a higher demand for Ca, and its bone resorption was more active, which may be susceptible to more bone loss (Dacke et al., 2015). We speculated that the AVS group was beneficial for both humerus and femur development compared to the CCS group, however, such differences may be weakened in the femur due to the differences of eggshell Ca supply.

Eggshell formation occurs primarily in the uterus. At least four Ca transporters (CALB, TRPV6, PMCA, NCX) have already been identified to be involved in the transcellular transport of Ca in the uterus (Bar, 2009). TRPV6 plays important roles in epithelial cellular entry for Ca, and there appeared to be a positive correlation between TRPV6 expression levels and Ca transport capacity (Yang et al., 2013). TRPV6 mRNA increased in the AVS group, which may indicate an enhanced Ca transport capacity, resulting in more Ca to enter the uterus. This could be an explanation for the increased content of eggshell Ca. The Ca transport in the intestinal tract primarily occurs in the anterior section of the small intestine and is considered to be dominated by transmembrane activities associated with CALB (Sugiyama et al., 2007). Herein, no differences were found at the mRNA expression level of CALB in the duodenum, hinting no significant differences in intestinal Ca absorption between the CCS and AVS groups.

Bone homeostasis requires the dynamic balance of bone formation and bone resorption (Feng and McDonald, 2011). Bone resorption could lead to bone mineral loss and damage to bone structure when bone homeostasis is out of the balance (Whitehead, 2004). However, this loss is required in some special physiological states, such as eggshell formation, whenever intestinal Ca absorption is insufficient. Bone resorption contributes 20%–40% of the eggshell Ca content and is one of the major means to supply eggshell Ca (Comar and Driggers, 1949; Clunies et al., 1993). TRAP has been recognized as a histochemical marker for osteoclasts more than several decades (Burstone, 1959). Although TRAP deletion did not affect osteoclast differentiation and resorption pits formation, it impaired the capacity of bone resorption (Hayman et al., 1996). The relative expression value of femur TRAP mRNA in the AVS group was significantly higher compared with the CCS group, which may indicate a higher bone resorption capacity for the femur in the AVS group. ALP and OCN are considered as markers for the early and late osteogenic stages, respectively (Farley et al., 1994; Xu et al., 2019). ALP plays a role in bone mineralization by promoting the hydroxyapatite growth (Nizet et al., 2020). OCN is involved in regulating the process of bone mineralization, and its upregulation promotes terminal osteogenic differentiation (Xu et al., 2019). The relative expression levels of femur ALP and OCN in the AVS group were also higher than those in the CCS group, suggesting the bone formation of the AVS group might be increased compared to the CCS group. Therefore, the increased expressions of TRAP, ALP and OCN indicated a higher bone remodeling of the femur in the AVS group, which may affect the Ca supply of eggshells, since bone resorption contributes 20%–40% of the eggshell Ca content and is one of the major means to supply eggshell Ca (Comar and Driggers, 1949; Clunies et al., 1993).

The RANK/RANKL/OPG pathway plays a dominant role in osteoclastogenesis. RANK is expressed on the osteoclast precursors, and its binding to RANKL activates NF-κB signaling in osteoclast precursors and induces osteoclasts maturation (Khosla, 2001). However, the decoy receptor OPG released by osteoblasts competes with RANK for binding to RANKL to block this process (Boyce and Xing, 2007). The ratio of RANKL/OPG is considered as a critical factor for osteoclast maturation and activation (Khosla, 2001; Dacke et al., 2015). Although the relative expression level of RANK in the CCS group was higher than in the AVS group, no significant differences were observed in the relative expression levels and rate of RANKL and OPG. Thus, there might be no differences between the CCS and AVS on the bone resorption of the humerus.

However, the humerus in the CCS group showed less bone formation. Active vitamin D and estrogen are involved in osteogenesis and bone formation after binding with the target receptor (mainly VDR and ER, respectively) (Hayashi et al., 2019; Chen et al., 2021). The deficiency of either of them accelerated bone loss and led to an OP phenotype (Chokalingam et al., 2012; Yang et al., 2020). Both VDR and ERα in the AVS group were higher expressed compared to those in the CCS group. In the AVS group, the increased expression of VDR and ERα could prevent bone loss (Peacock et al., 2002; Lam et al., 2014). On the contrary, the CCS group exhibited lower osteogenic ability and bone mass and was more prone to OP, which was consistent with the mechanical and compositional results. Similarly, the relative expression of FGF23 was also higher in the AVS group, and this result may be related to the change of VDR, since FGF23 is a phosphate regulator and involves a negative feedback regulation of vitamin D (Masuyama et al., 2006). Furthermore, the increased expression value of FGF23 may also be related to the increased Ca supply of the uterus in the AVS group. It has been reported that the constant stimulation of parathyroid hormone (PTH) secretion induced by the daily eggshell formation in the uterus could cause a chronic overexpression of FGF23 (Gloux et al., 2020). In our current study, the regulation of rearing systems on bone formation of humerus may be mainly through hormone-dependent pathways. Ca is precisely transmitted among the organs (i.e., the intestine, the kidney, the uterus and bone) by a complex network mediated by endocrine hormones, which mainly involves 1,25-dihydroxyvitamin D, PTH, and estrogen (Dacke et al., 2015). A previous study demonstrated that the effect of in-cage facilities on bone formation in laying hens was independent of PTH-related pathways (Dale et al., 2015). We found that the effect of rearing systems on the humerus may be related to 1,25-dihydroxyvitamin D and estrogen. Further studies will be necessary to determine the regulatory mechanism of the rearing systems on the secretion of 1,25-dihydroxyvitamin D and estrogen. It is also worth investigating whether the regulations of 1,25-dihydroxyvitamin D and estrogen can increase bone formation of the layers housed in the CCS.

In conclusion, compared with the CCS, the AVS alleviated the deterioration of eggshell and bone qualities of aged laying hens. The AVS upregulated the expression of genes associated with bone formation in the femur (ALP, OCN) and humerus (VDR, ERα and FGF23), which may be partly responsible for the improvement in bone quality. The AVS increased the expression of TRAP gene in the femur, hinting a higher bone resorption in the AVS, which may partly account for the improvement of eggshell quality.

## Data Availability

All data can be found in the article. The raw data supporting the conclusion of this article is available from the corresponding author upon reasonable request.
