# Supplementary material for: Effects of rearing systems on the eggshell quality, bone parameters and expression of genes related to bone remodeling in aged laying hens
Source: Front Physiol. 2022 Aug 31;13:962330. doi: 10.3389/fphys.2022.962330 (PMC9470921; doi:10.3389/fphys.2022.962330)
Supplement: Supplementary file 1 [file Table1.DOCX]

Supplementary Material

**Table S1** The composition and nutrient levels of the diets (air-dry basis)

|  | 9 – 18 weeks | 19 weeks - lay | lay - 55 weeks | 55 – 95 weeks |
| --- | --- | --- | --- | --- |
| Ingredient (%) | | | | |
| Corn | 68.00 | 63.00 | 58.40 | 63.00 |
| Soybean meal | 22.20 | 26.60 | 26.50 | 23.20 |
| Limestone | 1.25 | 4.70 | 9.00 | 9.90 |
| Dicalcium Phosphate | 1.40 | 1.20 | 1.20 | 1.20 |
| Salt | 0.36 | 0.36 | 0.36 | 0.36 |
| Soybean oil | 0.80 | 0.80 | 2.00 | - |
| Wheat bran | 3.75 | 1.10 | 0.30 | 0.10 |
| D,L-Methionine | 0.12 | 0.12 | 0.12 | 0.12 |
| Choline chloride | 0.12 | 0.12 | 0.12 | 0.12 |
| Premix | 2.00^1^ | 2.00^1^ | 2.00^2^ | 2.00^2^ |
| Total | 100.00 | 100.00 | 100.00 | 100.00 |
| Nutrient levels (calculated value) | | | | |
| AME (MJ/kg) | 11.75 | 11.50 | 11.29 | 10.89 |
| Crude protein (%) | 15.53 | 16.99 | 16.54 | 15.53 |
| Methionine (%) | 0.37 | 0.39 | 0.38 | 0.37 |
| Lysine (%) | 0.76 | 0.86 | 0.85 | 0.77 |
| Methionine + Cysteine (%) | 0.65 | 0.69 | 0.67 | 0.64 |
| Calcium (%) | 0.82 | 2.00 | 3.50 | 3.81 |
| Phosphorous (%) | 0.55 | 0.53 | 0.52 | 0.51 |
| Avaiable phosphorous (%) | 0.35 | 0.32 | 0.32 | 0.32 |

^1^ Premix provided the following per kg of the diet: vitamin A 6,000 IU; vitamin D_3_ 2,000 IU; vitamin E 15 IU; vitamin K 1.5 mg; thiamine 1.5 mg; riboflavin 6 mg; calcium pantothenate 25 mg; niacin 16 mg; pyridoxine 8 mg; biotin 0.5 mg; folic acid 1.25 mg; vitamin B_12_ 0.02mg; Mn 65 mg; I 1 mg; Fe 60 mg; Cu 8 mg; Zn 66 mg; montmorillonite 0.5 g; yeast culture 10 g.

^2^ Premix provided the following per kg of the diet: vitamin A 10,000 IU; vitamin D_3_ 4,125 IU; vitamin E 15 IU; vitamin K 2 mg; thiamine 1 mg; riboflavin 8.5 mg; calcium pantothenate 11 mg; niacin 32.5 mg; pyridoxine 8 mg; biotin 0.5 mg; folic acid 1.25 mg; vitamin B_12_ 0.02mg; Mn 65 mg; I 1 mg; Fe 60 mg; Cu 8 mg; Zn 66 mg; montmorillonite 1 g; yeast culture 10 g.

**Table S2** The primers used for qRT-PCR assays.

| Gene name | Primer sequence | Accession |
| --- | --- | --- |
| β-actin | F: 5’ TATGTGCAAGGCCGGTTTC 3’  R: 5’ TGTCTTTCTGGCCCATACCAA 3’ | NM_205518 |
| Runx2 | F: 5’ CACGCTGCTAAACCCAAACT 3’  R: 5’ GACTCATCCATCCTGCCACT 3’ | NM_001031102 |
| OCN | F: 5’ GAAGAGGCAGAAGAGGTTCG 3’  R: 5’ AGATAGTCACAGGGAGGGTAGC 3’ | XM_416184 |
| ALP | F: 5’ GGAGAAGGACCCCGAATACTG 3’  R: 5’ TTGACGCCGCAGAGGTAAG 3’ | NM_205360 |
| OPG | F: 5’ CGCTTGTGCTCTTGGACATT 3’  R: 5’ GCTGCTTTACGTAGCTCCCA 3’ | NM_001033641 |
| RANKL | F: 5’ AGGAGAAATAAGCCCGAGAA 3’  R: 5’ TTTGTTATGATGCCAGGATGTA 3’ | NM_001083361 |
| RANK | F: 5’ GGAGGGAAGGACACGGGAGTAC 3’  R: 5’ GCAGCACCGTCCAGAGTATTCATAG 3’ | XM_040666100 |
| TRAP | F: 5’ CTGGCTTTGGGCGATAACT 3’  R: 5’ TCGGAGTGTCGGCTGTATG 3’ | XM_040693093 |
| M-CSF | F: 5’ CAACAAACGCCAACAAGATG 3’  R: 5’ TCAAAGCACATTTCGGACAG 3’ | NM_001193295 |
| FGF23 | F: 5’ ATGCTGCTTGTGCTCTGTATC 3’  R: 5’ CACTGTAAATGGTTTGGTGAGG 3’ | XM_425663 |
| VDR | F: 5’ CCGGATTCAGGGATCTGACG 3’  R: 5’ AAGTCATTGCTTCCGCAGGT 3’ | NM_205098 |
| ERα | F: 5’ TGAGCTGGAGACTCTGAGCA 3’  R: 5’ CCGTACACTGGAGCGGTAGT 3’ | NM_205183 |
| ERβ | F: 5’ ATGACTTGCTGCTGGAGA 3’  R: 5’ CAGACCTGGAAATGTGAAAC 3’ | NM_204794 |
| CALB1 | F: 5’ TCTGGCACCACTACGACTCC 3’  R: 5’ GCCTTGCCATACTGGTCCAC 3’ | NM_205513 |
| NCX1 | F: 5’ TCCTAAGCCAGCAGCAGA 3’  R: 5’ ATGATGGTCAGGGCAACG 3’ | XM_015283438 |
| PMCA | F: 5’ TTACTGGTCTGACGTGCATTG 3’  R: 5’ AATCTTTGCCCTCCAAACAC 3’ | XM_015277056 |
| TRPV6 | F: 5’ TAAGACATTTGCCTGCCACA 3’  R: 5’ TTCAGCCCAGGAGTCAATCT 3’ | XM_040661661 |
